# Supplementary material for: Enhancing feelings of security: How institutional trust promotes interpersonal trust
Source: PLoS One. 2020 Sep 11;15(9):e0237934. doi: 10.1371/journal.pone.0237934 (PMC7486136; doi:10.1371/journal.pone.0237934)
Supplement: S1 File — (DOCX) [file pone.0237934.s001.docx]

**Supporting Information for:**

**“Enhancing feelings of security:**

**How institutional trust promotes interpersonal trust”**

Table of Contents

[S1 Appendix. Pilot Study 3](#_Toc47955904)

[S1 Text. Methods and results (Pilot Study) 3](#_Toc47955905)

[Text A. Manipulation of institutions (high vs. low presence) 8](#_Toc47955906)

[Text B. Framed trust game 8](#_Toc47955907)

[Table A. Descriptive statistics (Pilot Study). 9](#_Toc47955908)

[Table B. Results of the serial mediation models for the effect of the presence of institutions (manipulation) on trusting intentions through institutional trust (Pilot Study). 10](#_Toc47955909)

[S1 Table. Overview of the operationalization and descriptive statistics of the variables across the three studies. 11](#_Toc47955910)

[S2 Table. Correlations among scales of the survey (Study 1). 12](#_Toc47955911)

[S2 Text. Additional analyses (CFA) (Study 1). 13](#_Toc47955912)

[Table A. Goodness of fit models for Single Factor and Two Factors (Institutional trust and Feelings of security) (Study 1). 13](#_Toc47955913)

[S3 Table. Descriptive statistics (Study 2). 14](#_Toc47955914)

[S2 Appendix. Country-level covariates (Study 2). 15](#_Toc47955915)

[S4 Table. Results of multilevel mediation models for the effect of trust in different institutions on interpersonal trust through feelings of security (countries as random effects) (Study 2). 17](#_Toc47955916)

[S5 Table. Robustness check. Results of mediation models for the effect of trust in different institutions on interpersonal trust through feelings of security (countries as fixed effects) (Study 2). 18](#_Toc47955917)

[S3 Appendix. Manipulation of institutional trust (high vs. low) (Study 3) 20](#_Toc47955918)

[S6 Table. Descriptive statistics (Study 3). 22](#_Toc47955919)

[S7 Table. Results of mediation models for the effect of institutional trust (manipulation) on interpersonal trust through feelings of security (Study 3). 23](#_Toc47955920)

# S1 Appendix. Pilot Study

## S1 Text. Methods and results (Pilot Study)

Past research presents inconsistent findings on the effect of institutions on interpersonal trust, suggesting that institutions either undermine or sustain the development of trust among individuals [1]. However, our line of reasoning suggests that considering the extent those institutions are trusted can inform this debate [2]. To test this hypothesis, in this Pilot Study, we examined institutional trust as a mediator in the relationship between the presence of institutions and trusting beliefs and intentions.

**Method**

Eighty participants (45% females; *M*_age_ = 37.25 years, *SD*_age_ = 13.51) were recruited via social media posting to participate in an online experiment. Most had a high school diploma (58.8%) and described themselves as politically moderate on a 10-point political orientation scale ranging from left to right (*M* = 5.60, *SD* = 2.91). Sensitivity analysis revealed that this sample size would result in 80% statistical power to detect a medium effect of presence of institution on trust intentions (*d* = 0.64; [3]).

Participants were randomly assigned to one of the two experimental conditions manipulating the presence of institutions (low vs. high). They were asked to imagine visiting a fictitious foreign country and to read a travel guide to plan their stay. According to North’s definition of institutions as rules and procedures that structure human interaction [4], in the *high presence of institutions* condition, the country was described as highly regulated with many rules in place. Conversely, in the *low presence of institutions* condition, the participants read a description of a country governed by few and flexible regulations. Importantly, since we were interested in testing the effect of institutions *per se* on interpersonal trust, our manipulation did not include any cues about whether or not institutions could be trusted (see Text A in S1 Appendix). Four items were used as manipulation check (e.g., “*Many laws are in place*”, α = .91).

Then, participants completed a questionnaire that assessed trusting intentions and beliefs, institutional trust, security values, trust propensity, and included a brief socio-demographic section. Trusting intentions were assessed by asking participants to play a framed trust game [5] immediately after the manipulation. Participants were asked to imagine meeting a stranger once arrived at the train station, asking them for money with the promise of giving the entire amount back the following day in addition to a voucher of equivalent value (see Text B in S1 Appendix). Participants were then asked to indicate the amount of money that they would hypothetically lend the stranger (range: 0-20 €). This situation presents a real-world example of the most common games measuring trust [6].

Trusting beliefs regarding the stranger were assessed through an adaptation of the General Trust Scale ([7]; six items, e.g., “*I believe that this person is basically honest*”, α = .93). Similarly, we also assessed trusting beliefs toward the citizens of the country (six items, e.g., “*I believe that most citizens of Garovia will respond in kind when they are trusted by others”,* α = .93). Institutional trust was assessed adapting the reason-based trust scale [8] from tax authorities to public institutions (seven items, e.g., “*I trust public institutions in Garovia (e.g. ministries, police, government, etc.) because they behave benevolently towards citizens*”, α = .92). As control variables, we measured trust propensity through the Trust in Others Scale ([9]; three items, e.g., “*I dare to put my fate in the hands of most other people*”, α = .68) and the endorsement of security values using the respective subscale from the Portrait Values Questionnaire [10]. Here respondents are asked to indicate own perceived similarity to a described person (Five items, e.g., “*It is important to him to live in secure surroundings. He avoids anything that might endanger his safety*”, α = .76) on a six-point Likert scale from 1 (*Not like me at all*) to 6 (*Very much like me*).

**Results**

As expected, participants rated the fictitious country as significantly more regulated in the “high presence of institutions” condition (*M* = 5.73, *SD* = 1.14) compared to those in the “low presence of institutions” condition (*M* = 2.80, *SD*= 1.01), *t*(78) = -12.25, *p* < .001, *d* = 2.72), showing that participants perceived the manipulation as intended (see Table A in S1 Appendix).

**The main impact of institutions on interpersonal trust.** As predicted by the political-institutional perspective, participants perceived the stranger as more trustworthy in the high (*M* = 4.39, *SD* = 1.50) compared to the low presence of institutions condition (*M* = 3.74, *SD* = 1.29), *t*(78) = -2.08, *p* = .041, *d* = 0.46. Moreover, participants indicated a stronger intention to lend money to the stranger in the country with a high (*M* = 12.73, *SD* = 8.10) rather than a low presence of institutions (*M* = 7.17, *SD* = 7.02), *t*(78) = -3.29, *p* = .002, *d* = 0.74. No significant differences were found in trusting beliefs toward the citizens of the country between the two experimental conditions, *t*(78) = -0.58, *p* = .563, *d* = 0.13 (see Table A in S1 Appendix).

**The indirect effect of institutions on interpersonal trust via institutional trust.** We conducted a mediation analysis using the SPSS macro Process model 6 [11] with 5000 bootstrapped samples. As expected, the results of the serial multiple mediation analysis showed a significant effect of the presence of institutions on institutional trust, mediated by the trusting beliefs toward the stranger, which in turn impacted the money that the participants intended to lend. The full serial mediation model was significant, *b* = 1.33, 95% CI [0.35; 2.49], *R*^2^ = .38.

To be consistent with previous studies (i.e., [12]), we additionally tested this hypothesis considering trusting beliefs toward the local society as dependent measure. Consistent with the above-mentioned findings, institutions elicited institutional trust, which in turn affected the trusting beliefs towards the citizens, and trusting intentions. The full serial mediation model was significant, *b* = 0.95, 95% CI [0.20; 1.89], *R*^2^ = .21). The expected serial mediation also remained significant if individual characteristics (i.e., trust propensity, endorsement of security values, and political orientation) are included as covariates: trusting beliefs toward the stranger, *b* = 1.01; 95% CI [0.22; 1.95], *R*^2^ = .40, and the citizens, *b* = 0.70; 95% CI [0.09; 1.52], *R*^2^ = .25. All path coefficients are presented in detail in Table B in S1 Appendix.

**Discussion**

In line with the political-institutional perspective, the results of the Pilot Study showed that the presence of institutions directly enhanced participants’ trusting beliefs and intentions towards a single unknown person (i.e., the willingness to lend money to a stranger). Moreover, we found that trust in these institutions, in turn, predicted both interpersonal trust toward the stranger and citizens. Results also showed that individuals’ trust propensity, security values, and political attitudes did not influence this effect. Remarkably, trusting beliefs and intentions toward the stranger increased even if the institutions were manipulated in a scenario setting in which they had no actual power to influence the stranger’s decision to honour or betray trust.

**References**

1. Robbins BG. Neither government nor community alone: A test of state-centered models of generalized trust. Ration Soc. 2011;23: 304–346. doi:10.1177/1043463111404665

2. Baldassarri D, Grossman G. Centralized sanctioning and legitimate authority promote cooperation in humans. Proc Natl Acad Sci U S A. 2011;108: 11023–11027. doi:10.1073/pnas.1105456108

3. Faul F, Erdfelder E, Lang AG, Buchner A. G*Power 3: A flexible statistical power analysis program for the social, behavioral, and biomedical sciences. Behavior Research Methods. Psychonomic Society Inc.; 2007. pp. 175–191. doi:10.3758/BF03193146

4. North DC. Institutions, Institutional Change and Economic Performance. Cambridge: Cambridge University Press; 1990. Available: https://books.google.com/books?hl=it&lr=&id=oFnWbTqgNPYC&pgis=1

5. Berg J, Dickhaut J, McCabe K. Trust, reciprocity, and social history. Games Econ Behav. 1995;10: 122–142. doi:10.1006/game.1995.1027

6. Evans AM, Krueger JI. The Psychology (and Economics) of Trust. Soc Personal Psychol Compass. 2009;3: 1003–1017. doi:10.1111/j.1751-9004.2009.00232.x

7. Yamagishi T, Yamagishi M. Trust and commitment in the United States and Japan. Motiv Emot. 1994;18: 129–166. doi:10.1007/BF02249397

8. Hofmann E, Gangl K, Kirchler E, Stark J. Enhancing tax compliance through coercive and legitimate power of tax authorities by concurrently diminishing or facilitating trust in tax authorities. Law Policy. 2014;36: 290–313. doi:10.1111/lapo.12021

9. Van Lange PAM, Vinkhuyzen AAE, Posthuma D. Genetic influences are virtually absent for trust. PLoS One. 2014;9. doi:10.1371/journal.pone.0093880

10. Schwartz SH, Melech G, Lehmann A, Burgess S, Harris M, Owens V. Extending the Cross-Cultural Validity of the Theory of Basic Human Values with a Different Method of Measurement. J Cross Cult Psychol. 2001;32: 519–542. doi:10.1177/0022022101032005001

11. Hayes AF. Introduction to mediation, moderation, and conditional process analysis: A regression-based approach. New York: Guilford Press; 2013.

12. Rothstein B, Eek D. Political Corruption and Social Trust. Ration Soc. 2009;21: 81–112. doi:10.1177/1043463108099349

## Text A. Manipulation of institutions (high vs. low presence)

Imagine you are traveling.
Your destination is a country called Garovia. Since you are visiting this country for the first time and you are going to live there for some months, you decide to check a tourist guide containing useful information for your journey in order to plan your stay.
So, you start to read the first section of the guide, and concerning some characteristics of the country linked to its administration you read the following:

“Garovia is known for its proud population. It is a country with many/few public institutions and in, this country, order represents an issue of primary/secondary importance. Consequently, public life in Garovia is regulated by many/few formal rules and regulations. For example, when you walk through the streets or public areas (as parks, squares or gardens), you will always/never see signs that show which behaviors are encouraged or forbidden. All existing rules are handled in a strict/flexible way. Generally, Garovia can be described as a country with many/few regulations.”

## Text B. Framed trust game

Once arrived at the station in the afternoon, you start to walk with no haste towards to your destination while still thinking about the information you have just read on the guide.

At one point, you are stopped by a stranger, who introduces himself with the unique Garovian accent. He asks you to lend him the equivalent of 20 euros in national currency, explaining that due to the haste of arriving on time to the station before the final run of the train, he has forgotten his wallet with the train ticket reservations and other useful tickets inside.

He tells you that in this moment any amount of money would help him and that he will give the money back to you the following day, setting up an appointment at the station.

Furthermore, he adds that in exchange for your availability, he intends to bring you a voucher of equivalent value, expendable in a supermarket chain present in the whole nation.

In this moment, you have the amount of money that that person is asking for, and the station is easy to reach from your residence.

You think for a while and…

## **Table A. Descriptive statistics (Pilot Study).**

|  | **Presence of Institutions** | |
| --- | --- | --- |
|  | **Low** | **High** |
|  | **M (SD)** | **M (SD)** |
| Trusting beliefs (toward the stranger) * | 3.74 (1.29) | 4.39 (1.50) |
| Trusting beliefs (toward citizens) | 4.45 (0.93) | 4.59 (1.24) |
| Trusting intentions (money range 0 - 20) ** | 7.17 (7.02) | 12.73 (8.10) |
| Institutional trust ** | 3.87 (1.27) | 4.72 (1.05) |

*M* = mean, *SD* = standard deviation, **p* < .05, ***p* < .01, ****p* < .001

## Table B. Results of the serial mediation models for the effect of the presence of institutions (manipulation) on trusting intentions through institutional trust (Pilot Study).

| **Predictor and effect** | **Outcome: trusting intentions toward the stranger** | | | | | |
| --- | --- | --- | --- | --- | --- | --- |
|  | **Model 1** | | | **Model 2** | | |
|  | ***b*** | ***SE*** | **95% CI** | ***b*** | ***SE*** | **95% CI** |
| Mediator1: Institutional trust* | -1.18 | 0.69 | -2.56; 0.19 | -1.13 | 0.70 | -2.52; 0.27 |
| Mediator2: Trusting beliefs toward the stranger* | 3.20 | 0.56 | 2.08; 4.32 | 3.14 | 0.61 | 1.93; 4.35 |
| *Presence of institutions (Manipulation)* |  | | |  | | |
| Total effect | 5.56 | 1.72 | 2.15; 8.98 | 5.89 | 1.70 | 2.51; 9.28 |
| Direct effect | 4.49 | 1.56 | 1.39; 7.60 | 4.80 | 1.58 | 1.64; 7.95 |
| Indirect effect | 1.33 | 0.55 | 0.35; 2.49 | 1.01 | 0.44 | 0.22; 1.95 |
| Mediator1: Institutional trust* | -0.75 | 0.82 | -2.37; 0.88 | -0.75 | 0.82 | -2.38; 0.87 |
| Mediator2: Trusting beliefs toward the citizens* | 2.44 | 0.85 | 0.76; 4.13 | 2.20 | 0.89 | 0.43; 3.98 |
| *Presence of institutions (Manipulation)* |  | | |  | | |
| Total effect | 5.56 | 1.72 | 2.15; 8.98 | 5.89 | 1.7 | 2.51; 9.28 |
| Direct effect | 5.85 | 1.77 | 2.33; 9.38 | 6.26 | 1.77 | 2.73; 9.80 |
| Indirect effect | 0.95 | 0.44 | 0.20; 1.89 | 0.70 | 0.37 | 0.09; 1.52 |

Results based on 80 observations. Model 1: Mediation analyses did not include control variables. Model 2: Mediation analyses included trust propensity, security values, and political orientation as control variables. *Estimates of regressions of the mediators (institutional trust and trusting beliefs, respectively) predicting trusting intentions.

# S1 Table. Overview of the operationalization and descriptive statistics of the variables across the three studies.

| **Study #** | **Variable** | **Operationalization** | **Cronbach's α** | **M** | **SD** |
| --- | --- | --- | --- | --- | --- |
| **Study 1** | Institutional trust | Trust perception toward five institutions (police, legal system, government, media, religious institutions). Each of them assessed through an adaptation of the Reason-based Trust Scale (Hofmann et al., 2014) | 0.70 | 3.35 | 0.82 |
|  | Feelings of security | Feelings of security experienced in relation to institutions. Assessed through a self-created 3-items scale | 0.89 | 2.65 | 1.29 |
|  | Trusting beliefs | Trust perception toward Italian citizens. Assessed through an adaptation of the General Trust Scale (Yamagishi & Yamagishi, 1994) | 0.93 | 3.66 | 1.15 |
| **Study 2** | Institutional trust | Trust perception toward four institutions (parliament, legal system, police, politicians). Each of them assessed through a single ESS item | 0.78-0.87 | 3.54-6.73 | 1.58-2.15 |
|  | Feelings of security | Perception of personal safety in local areas. Assessed through a single ESS item | - | 2.83-3.41 | 0.61-0.92 |
|  | Trusting beliefs | Trust perception toward most people. Assessed through a single ESS item | - | 4.20-6.82 | 1.42-1.97 |
| **Study 3** | Institutional trust | Manipulation of the information (i.e., competence, benevolence, and reliability) provided to participants about police in partner's country in the trust game | - | - | - |
|  | Feelings of security | Feelings of security experienced in relation to the police of partner's country in the trust game. Assessed through a 3-items scale | 0.96 | 2.50 (4.67) | 1.42 (1.28) |
|  | Expectations of reciprocity | Percentage of money expected to receive back from the partner | - | 45.98 (52.02) | 21.22 (20.80) |
|  | Trusting beliefs | Trust perception toward the partner in the trust game. Assessed through an adaptation of the General Trust Scale (Yamagishi & Yamagishi, 1994) | 0.88 | 4.76 (4.93) | 0.78 (0.89) |
|  | Trusting behavior | Money transferred to the partner in the trust game | - | 3.39 (3.67) | 1.39 (1.27) |

In Study 2, lower and upper range values found across countries are provided. In Study 3, mean and standard deviation in brackets refer to the high institutional trust condition.

# S2 Table. Correlations among scales of the survey (Study 1).

|  | **M (SD)** | **1** | **2** | **3** | **4** | **5** | **6** | **7** | **8** | **9** | **10** |
| --- | --- | --- | --- | --- | --- | --- | --- | --- | --- | --- | --- |
| **1. Trust in institutions (aggregate)** | 3.35 (0.82) |  |  |  |  |  |  |  |  |  |  |
| **2. Trust in police** | 3.56 (1.11) | .72*** |  |  |  |  |  |  |  |  |  |
| **3. Trust in legal system** | 3.43 (1.19) | .72*** | .42*** |  |  |  |  |  |  |  |  |
| **4. Trust in government** | 2.67 (1.09) | .74*** | .44*** | .48*** |  |  |  |  |  |  |  |
| **5. Trust in media** | 3.37 (1.06) | .50*** | .30*** | .38*** | .40*** |  |  |  |  |  |  |
| **6. Trust in religious institutions** | 3.59 (1.37) | .61*** | .26*** | .16* | .22** | .28*** |  |  |  |  |  |
| **7. Feelings of security** | 2.65 (1.29) | .50*** | .42*** | .50*** | .43*** | .27*** | .14 |  |  |  |  |
| **8. Trusting beliefs** | 3.67 (1.15) | .28** | .24** | .23** | .23** | .19** | .15 | .33*** |  |  |  |
| **9. Trust propensity** | 3.41 (1.33) | .11 | .01 | .13 | .12 | .06 | .08 | .22** | .39*** |  |  |
| **10. Security values** | 3.99 (0.90) | .22** | .28*** | .01 | .04 | .30*** | .27*** | .01 | .05 | -.21** |  |
| **11. Political orientation** | 4.06 (2.31) | 0.08 | .27*** | -.16* | -.06 | .17* | .21** | -.01 | .04 | -.18* | .36*** |

*M* = mean, *SD* = standard deviation. **p* < .05, ***p* < .01, ****p* < .001

# S2 Text. Additional analyses (CFA) (Study 1).

Given that the strong correlation between institutional trust and feelings of security (*r* = .50) suggests a possible partial overlap between the institutional trust and feelings of security, we run two confirmatory factor analyses (CFA) with a maximum likelihood estimation to compare the model fit of either one or two latent factors using *lavaan* version 0.5-23 [1] in R version 3.4.2 [2]. Results showed an initial evidence of a better fit of the two-factors model (considering the feelings of security and institutional trust as separate factors), compared to the one-factor solution.

## Table A. Goodness of fit models for Single Factor and Two Factors (Institutional trust and Feelings of security) (Study 1).

| **Model** | **CFI** | **SRMR** | **BIC** | **AIC** |
| --- | --- | --- | --- | --- |
| Single Factor | 0.76 | 0.109 | 5214.28 | 5213.71 |
| Two Factors | 0.928 | 0.06 | 4996.1 | 4995.51 |

**References**

1. Rosseel Y. Lavaan: An R package for structural equation modeling. J Stat Softw. 2012;48: 1–36. doi:10.18637/jss.v048.i02

2. R Core Team. R: A Language and Environment for Statistical Computing. Vienna, Austria; 2019. Available: https://www.r-project.org

# S3 Table. Descriptive statistics (Study 2).

| **Country** | **N*_initial_*** | **N*_final_*** | **% F** | **M*_age_*(SD)** |
| --- | --- | --- | --- | --- |
| Belgium | 12577 | 11560 | 49.96 | 45.81(18.41) |
| Switzerland | 12335 | 10123 | 50.01 | 47.98(17.83) |
| Germany | 20490 | 17897 | 48.19 | 48.08(17.72) |
| Denmark | 10836 | 9334 | 47.38 | 48.01(17.62) |
| Spain | 13543 | 10934 | 48.7 | 45.15(17.67) |
| Finland | 14275 | 13424 | 50.46 | 47.64(18.47) |
| France | 12981 | 12004 | 52.77 | 48.68(18.08) |
| United Kingdom | 15667 | 12604 | 52.83 | 49.35(18.26) |
| Hungary | 11518 | 8888 | 53.08 | 46.73(17.74) |
| Ireland | 15490 | 12346 | 52.61 | 47.03(17.62) |
| The Netherlands | 13505 | 12089 | 53.35 | 49.33(17.29) |
| Norway | 11703 | 9937 | 45.67 | 45.92(17.36) |
| Poland | 12430 | 9436 | 49.52 | 42.13(17.84) |
| Portugal | 13718 | 10859 | 56.49 | 49.36(18.92) |
| Sweden | 12839 | 10755 | 47.96 | 47.48(18.25) |
| Slovenia | 9607 | 7861 | 51.46 | 45.27(18) |
| Total | 213514 | 180051 |  |  |

Sample size (initial and final), female proportion and mean age for each sample of the 16 countries included from the ESS. The final sample sizes include only participants not presenting missing answers on the variables: institutional trust, interpersonal trust, feelings of security, gender, age, and education.

# S2 Appendix. Country-level covariates (Study 2).

***Political indicators***

**Government effectiveness.** The Worldwide Government Indicator project (WGI; [1]) has reported aggregate indicators measuring several dimensions of governance, including government effectiveness (i.e., the ability of government in providing high quality public services and to implement effective policies) over 200 countries. In this study, we included government effectiveness average scores for the 16 countries of reference between 2002 and 2014. Values range from -2.5 to 2.5, with higher scores indicating stronger government effectiveness.

**Political rights.** Freedom House’s Political Rights Index [2] has been used to assess the degree to which each of the selected country enjoys a variety of political rights. Among them, the fairness and accountability of the electoral process, the good representation of all social groups in politics, and government accountability. Average scores from 2003 to 2014 have been used, ranging from 0 to 40, with higher scores indicating higher levels of political rights.

**Rule of law.** The index of rule of law provided by the Worldwide Government Indicator project (WGI; [1]) has been used in this study to capture the extent by which agents perceive that each country has a “law and order tradition”. It makes reference to confidence in the state’s ability to protect legal entitlements, and to maintain social order through formal rules. We selected country average indexes between 2002 and 2014, ranging from -2.5 to 2.5, with higher scores indicating stronger rule of law.

***Economic indicators***

**Economy competitiveness.** Global Competitiveness Index (GCI; [3]) released by World Economic Forum has been used to capture the level of competitiveness of each country’s economy, defined as the level of economical productivity and prosperity attainable by a country given its institutions. In this study, we included scores from the 2014-2015 edition, ranging from 1 to 7, with higher scores indicating greater competitiveness.

**GINI.** Gini coefficient was used as measure of income inequality. Country coefficients from years 2007. 2008, 2010, and 2011 were taken from World Bank’s database [4] and averaged, with values ranging from 0 to 100, with higher scores indicating perfect inequality.

**GDP per capita.** We used levels of gross domestic product (GDP) per capita, adjusted for purchasing power parity (PPP) to capture each country’s economic wealth. The data are selected from the World Economic Outlook of the International Monetary Fund [5]. In the current study, GDP indexes were used by averaging those obtained from 2002 to 2014.

**References**

1. Kaufmann D, Kraay A, Mastruzzi M. The worldwide governance indicators: Methodology and analytical issues. Hague J Rule Law. 2011;3: 220–246. doi:10.1017/S1876404511200046

2. Freedom House. Freedom in the World 2015. 2015 [cited 2 Dec 2019]. Available: http://www.freedomhouse.org/report/freedom-world/freedom-world-2015

3. Schwab K. The Global Competitiveness Report 2014–2015: Full Data Edition. 2014.

4. World Bank. World Development Indicators. GINI index (World Bank Estimate). 2011 [cited 12 Jan 2019]. Available: http://data.worldbank.org/indicator/SI.POV.GINI

5. World Economic Outlook of the International Monetary Fund. World Economic Outlook Database. 2017 [cited 12 Dec 2019]. Available: https://www.imf.org/external/pubs/ft/weo/2017/01/weodata/download.aspx

# S4 Table. Results of multilevel mediation models for the effect of trust in different institutions on interpersonal trust through feelings of security (countries as random effects) (Study 2).

| **Predictor and effect** | **Model 1** | | **Model 2** | | **Model 3** | |
| --- | --- | --- | --- | --- | --- | --- |
|  | ***b*** | **95% CI** | ***b*** | **95% CI** | ***b*** | **95% CI** |
| Mediator: Feelings of security | 0.338 | 0.3377; 0.3383 | 0.370 | 0.3700; 0.3705 | 0.335 | 0.3354; 0.3363 |
| *Predictor: Institutional trust (Parliament)* |  | |  | |  | |
| Total effect | 0.226 | 0.2232; 0.2291 | 0.218 | 0.2148; 0.2221 | 0.227 | 0.2240; 0.2302 |
| Direct effect | 0.216 | 0.2131; 0.2188 | 0.210 | 0.2068; 0.2136 | 0.217 | 0.2138; 0.2201 |
| Indirect effect | 0.010 | 0.0095; 0.0112 | 0.008 | 0.0079; 0.0091 | 0.010 | 0.0095; 0.0111 |
| % of Total effect | 0.05 | | 0.04 | | 0.05 | |
| Mediator: Feelings of security | 0.316 | 0.2865; 0.3450 | 0.327 | 0.3268; 0.3274 | 0.293 | 0.2930; 0.2939 |
| *Predictor: Institutional trust (Legal system)* |  | |  | |  | |
| Total effect | 0.222 | 0.2190; 0.2256 | 0.215 | 0.2115; 0.2178 | 0.222 | 0.2186; 0.2253 |
| Direct effect | 0.211 | 0.2083; 0.2144 | 0.205 | 0.2015; 0.2081 | 0.211 | 0.2083; 0.2139 |
| Indirect effect | 0.011 | 0.0100; 0.0117 | 0.010 | 0.0089; 0.0106 | 0.011 | 0.0099; 0.0118 |
| % of Total effect | 0.05 | | 0.05 | | 0.05 | |
| Mediator: Feelings of security | 0.296 | 0.2967; 0.2974 | 0.316 | 0.3162; 0.3168 | 0.295 | 0.2948; 0.2958 |
| *Predictor: Institutional trust (Police)* |  | |  | |  | |
| Total effect | 0.213 | 0.2092; 0.2157 | 0.205 | 0.2026; 0.2086 | 0.212 | 0.2080; 0.2152 |
| Direct effect | 0.205 | 0.2015; 0.2079 | 0.197 | 0.1941; 0.2004 | 0.204 | 0.2008; 0.2072 |
| Indirect effect | 0.007 | 0.0068; 0.0087 | 0.008 | 0.0076; 0.0091 | 0.008 | 0.0069; 0.0084 |
| % of Total effect | 0.04 | | 0.04 | | 0.04 | |
| Mediator: Feelings of security | 0.319 | 0.3186; 0.3191 | 0.339 | 0.3389; 0.3393 | 0.315 | 0.3148; 0.3155 |
| *Predictor: Institutional trust (Politicians)* |  | |  | |  | |
| Total effect | 0.251 | 0.2479; 0.2547 | 0.245 | 0.2422; 0.2475 | 0.252 | 0.2477; 0.2551 |
| Direct effect | 0.243 | 0.2403; 0.2470 | 0.238 | 0.2351; 0.2401 | 0.244 | 0.2404; 0.2476 |
| Indirect effect | 0.008 | 0.0069; 0.0083 | 0.007 | 0.0068; 0.0080 | 0.007 | 0.0068; 0.0081 |
| % of Total effect | 0.03 | | 0.03 | | 0.03 | |

Results of all models are based on 180,051 observations and use countries as random effects. % of Total effect: Proportion mediated (i.e., ratio of the total effect to the indirect effect).

Model 1: Mediation analyses included survey wave as control variable. Model 2: Mediation analyses included survey wave and individual-level variables (gender, age, and education) as control. Model 3: Mediation analyses included survey wave and country-level variables (political rights, government effectiveness, rule of law, economy competitiveness, GINI coefficient, and GDP per capita) as control.

# S5 Table. Robustness check. Results of mediation models for the effect of trust in different institutions on interpersonal trust through feelings of security (countries as fixed effects) (Study 2).

| **Predictor and effect** | **Model 1** | | **Model 2** | |
| --- | --- | --- | --- | --- |
|  | ***b*** | **95% CI** | ***b*** | **95% CI** |
| Mediator: Feelings of security | 0.298 | 0.274; 0.3214 | 0.326 | 0.3026; 0.3495 |
| *Predictor: Institutional trust (Aggregate)* |  | |  | |
| Total effect | 0.352 | 0.3473; 0.3552 | 0.342 | 0.3370; 0.3459 |
| Direct effect | 0.338 | 0.3338; 0.3417 | 0.329 | 0.3244; 0.3330 |
| Indirect effect | 0.013 | 0.0125; 0.0144 | 0.013 | 0.01175; 0.0137 |
| % of Total effect | 0.04 | | 0.04 | |
| Mediator: Feelings of security | 0.338 | 0.3152; 0.3603 | 0.370 | 0.3475; 0.3925 |
| *Predictor: Institutional trust (Parliament)* |  | |  | |
| Total effect | 0.226 | 0.2229; 0.2299 | 0.218 | 0.2151; 0.2214 |
| Direct effect | 0.216 | 0.2139; 0.2193 | 0.210 | 0.2066; 0.2131 |
| Indirect effect | 0.010 | 0.0097; 0.0111 | 0.008 | 0.0078; 0.0092 |
| % of Total effect | 0.05 | | 0.04 | |
| Mediator: Feelings of security | 0.295 | 0.2705; 0.3187 | 0.327 | 0.3028; 0.3509 |
| *Predictor: Institutional trust (Legal system)* |  | |  | |
| Total effect | 0.222 | 0.2189; 0.2251 | 0.215 | 0.2117; 0.2180 |
| Direct effect | 0.211 | 0.2082; 0.2143 | 0.205 | 0.2021; 0.2081 |
| Indirect effect | 0.011 | 0.0010; 0.0118 | 0.010 | 0.0089; 0.0105 |
| % of Total effect | 0.05 | | 0.05 | |
| Mediator: Feelings of security | 0.295 | 0.2948; 0.2958 | 0.316 | 0.2875; 0.3450 |
| *Predictor: Institutional trust (Police)* |  | |  | |
| Total effect | 0.212 | 0.2085; 0.2151 | 0.206 | 0.2023; 0.2089 |
| Direct effect | 0.204 | 0.2013; 0.2075 | 0.198 | 0.1944; 0.2004 |
| Indirect effect | 0.008 | 0.0069; 0.0085 | 0.008 | 0.0072; 0.0093 |
| % of Total effect | 0.04 | | 0.04 | |
| Mediator: Feelings of security | 0.319 | 0.298; 0.3391 | 0.339 | 0.3183; 0.3594 |
| *Predictor: Institutional trust (Politicians)* |  | |  | |
| Total effect | 0.251 | 0.2472; 0.2535 | 0.245 | 0.2417; 0.2482 |
| Direct effect | 0.243 | 0.2401; 0.2459 | 0.238 | 0.2341; 0.2410 |
| Indirect effect | 0.008 | 0.0068; 0.0083 | 0.007 | 0.0066; 0.0081 |
| % of Total effect | 0.03 | | 0.03 | |

Results of all models are based on 180,051 observations and use countries as fixed effects. % of Total effect: Proportion mediated (i.e., ratio of the total effect to the indirect effect).

Model 1: Mediation analyses included country and survey wave as control variable. Model 2: Mediation analyses included country, survey wave, and individual-level variables (gender, age, and education) as control. Model 3: Mediation analyses included country, survey wave and country-level variables (political rights, government effectiveness, rule of law, economy competitiveness, GINI coefficient, and GDP per capita) as control.

# S3 Appendix. Manipulation of institutional trust (high vs. low) (Study 3)

IEA (Institute of Economic Affairs) has recently published the report of data collected in a 2014 Survey on institutional performance of Country X.

Second player comes from a country in which…

Police represent 4% of the labor force of Country X.

According to the citizens’ perception, police have a good/bad reputation.

Compared to international standards, police officers receive a highly appropriate/inappropriate training, which throughout the years results in the acquisition/lack of professional skills necessary to respond properly to citizens’ needs.

According to the statistics, police seem to/ to not fulfill their obligations in accordance to the goals set by the Annual Plan for Crime Reduction.

The use of power in this position is always/not always handled responsibly.

At the question “Do you feel you can rely on police in your country?” 85% of respondent answered “absolutely yes/no”, while just 5% answered “absolutely no/yes”.

As trends of last decade show, there have/have not been many documented cases of police officers who do not follow State Police Code of Conduct. This year, the number of police officers removed from their job for infringement of Code rules has reached the minimum/maximum in ten years.

In general, according to citizens’ perception, the police in their country seems to/does not seem to work well.

Moreover, it is a diffused perception among citizens that police officers put the societal interests/ only few people's interests first in the way they operate.

These trends are in line with the ones shown by CPI (an international perceived corruption index), which reports Country X among one of the 5 least/most corrupt countries considered in the survey.

At the question “Do you generally trust police in your country?” 85% of respondent answered “absolutely yes/no”, while just 5% answered “absolutely no/yes”.

# S6 Table. Descriptive statistics (Study 3).

|  | **Institutional Trust** | |
| --- | --- | --- |
|  | **Low** | **High** |
|  | **M (SD)** | **M (SD)** |
| Trusting beliefs | 4.76 (0.78) | 4.93 (0.89) |
| Trusting behavior | 3.39 (1.39) | 3.67(1.28) |
| Expectations of reciprocity | 0.46 (0.21) | 0.52 (0.21) |
| Feelings of security *** | 2.50 (1.42) | 4.67 (1.28) |

*M* = mean, *SD* = standard deviation. **p* < .05, ***p* < .01, ****p* < .001

# S7 Table. Results of mediation models for the effect of institutional trust (manipulation) on interpersonal trust through feelings of security (Study 3).

| **Predictor and effect** | **Outcome: Trusting beliefs** | | | | | |
| --- | --- | --- | --- | --- | --- | --- |
|  | **Model 1** | | | **Model 2** | | |
|  | ***b*** | ***SE*** | **95% CI** | ***b*** | ***SE*** | **95% CI** |
| Mediator: Feelings of security | 0.19 | 0.06 | 0.06; 0.31 | 0.17 | 0.06 | 0.04; 0.29 |
| *Institutional trust* |  | | |  | | |
| Total effect | 0.17 | 0.17 | -0.17; 0.52 | 0.33 | 0.17 | -0.01; 0.66 |
| Direct effect | -0.26 | 0.22 | -0.69; 0.17 | -0.07 | 0.22 | -0.50; 0.36 |
| Indirect effect | 0.41 | 0.17 | 0.13; 0.83 | 0.37 | 0.18 | 0.06; 0.80 |
| Mediator: Expectations of reciprocity | 0.01 | 0.06 | 0.06; 0.31 | 0.01 | 0.01 | -0.02; 0.01 |
| *Institutional trust* |  | | |  | | |
| Total effect | 0.17 | 0.17 | -0.17; 0.52 | 0.33 | 0.17 | -0.01; 0.66 |
| Direct effect | -0.26 | 0.22 | -0.69; 0.17 | -0.07 | 0.22 | -0.50; 0.36 |
| Indirect effect | 0.02 | 0.03 | -0.01; 0.15 | 0.02 | 0.03 | -0.01; 0.14 |

Results based on 94 observations. Model 1: Mediation analyses did not include control variables. Model 2: Mediation analyses included trust propensity, security values, political orientation, risk attitudes, and education as control variables.
