# Supplementary material for: Enhancing feelings of security: How institutional trust promotes interpersonal trust
Source: PLoS One. 2020 Sep 11;15(9):e0237934. doi: 10.1371/journal.pone.0237934 (PMC7486136; doi:10.1371/journal.pone.0237934)
Supplement: Attachment — Submitted filename: Review_plos.pdf [file pone.0237934.s004.pdf]

This is, potentially, an interesting contribution to the literature on institutional and social trust. However, the manuscript has several issues and requires major revisions to be publishable. Here are some suggestions to improve the paper:

General

1. The authors argue that the relationship between social and political trust has been neglected in the literature (e.g. in the conclusions: "Decades of research have focused on several processes that may promote trust among strangers, but very little attention has been devoted on one recurrent feature that characterize modern human interactions: the presence of institutions"). However, as the authors acknowledge in their literature review, there is a relevant body of research investigating precisely this relationship following different approaches: Sonderskov, Brehm & Rahn, Lekti, Rothstein, Uslaner, Stolle have addressed this topic (all mentioned in the manuscript). I would suggest to add the following references as well:

Herreros F, Criado H. The state and the development of social trust. International Political Science Review. 2008;29(1):53–71
Lo Iacono S. (2019). Law-breaking, fairness, and generalized trust: The mediating role of trust in institutions. PloS one, 14(8).
Richey S. The impact of corruption on social trust. American Politics Research. 2010;38(4):676–90.
Js You. Social trust: Fairness matters more than homogeneity. Political Psychology. 2012;33(5):701–21.

I invite the authors to acknowledge previous research on institutional trust/institutions and social trust throughout the entire manuscript (in line with their literature review), while fleshing out more clearly the main contribution of the manuscript, namely the analysis of the mediation effect and the disentangling of the psychological processes behind the relationship (which, indeed, has not been empirically investigated, though theoretically argued to some extent – e.g. Rothstein and Stolle 2008).

2. The methods and results sections need extensive revising for all three studies. Given the wide variety of measures employed, it is often unclear how concepts are operationalized. A descriptive table showing the coding, the mean, SD, N of the variables used in each study would be extremely helpful (maybe you can include this in the SI). Also, it would be good to present results from the mediation models in a table (one for each study), showing the effects with and without covariates (in the SI you could report the complete tables with all coefficients). Side note: at p. 15 the authors mention that "the lack of control for individual differences in previous cross-sectional studies might have overestimated the relationship between the two forms of trust in the past". Looking at previous studies in the literature (see point 1), this is hardly correct (they do employ a wide variety of controls at the individual level). Also, while using controls in Studies 1 and 2 makes perfect sense because they are observational studies, Study 3 is an experiment and it shouldn't require controls if the randomization worked properly. Including controls for Study 3 should be justified by arguing that you are adjusting for (potential) differences in baseline covariates across the different conditions.

3. There is no explanation or description of the pilot in the main text – the authors briefly mentioned it in the literature review at p. 11: "Thus, for a better understanding of the relation between institutions and interpersonal trust, in the next section, we will focus on research addressing the effect of institutional trust on interpersonal trust. In S1 Appendix we report the results of an additional

experimental pilot study that manipulated the presence vs. absence of institutions, and provided evidence for its cascading effects on institutional trust, trusting beliefs, and trusting behavioral intentions towards a stranger". I would suggest the authors to provide a more detailed justification of the pilot in the general description of their work.

4. The manuscript requires a careful revision of the text. Sometimes sentences appear quite disconnected, or are simply inconsistent with the rest of the paragraph. I report here a couple of cases, but an accurate double-check is required. Examples, p.15: "The whole research was conducted in accordance with the Declaration of Helsinki (7th revision, 2013) and local ethical guidelines for experimentation with human participants and was approved by the institutional review board at the University of Turin and by the ethical commission of the Zeppelin University in Friedrichshafen. All subjects gave written informed consent prior to the experiment. To avoid sequence effects, in Studies 1 and 3 all items were presented in a randomized order within each scale and, unless otherwise stated, they were answered on a seven-point Likert scale from 1 (I do not agree) to 7 (I totally agree)".
At p. 17 (while describing Study 1): "From these items, we created a general aggregated measure of institutional trust by averaging scores of these five scales. As in Study 1, trusting beliefs toward Italian citizens were measured through the adapted General Trust Scale ([23], $\alpha = .93$)".

5. In the manuscript, the authors often discuss the direct impact of institutional trust on interpersonal trust. Fig. 1, however, does not represent this accurately (as it shows only an effect on trusting behavior). Thus, I would suggest to modify Fig.1 and make it more consistent with the authors' general argument/interpretation of results. Here is a possible "solution" (apologies for the sketchy picture):

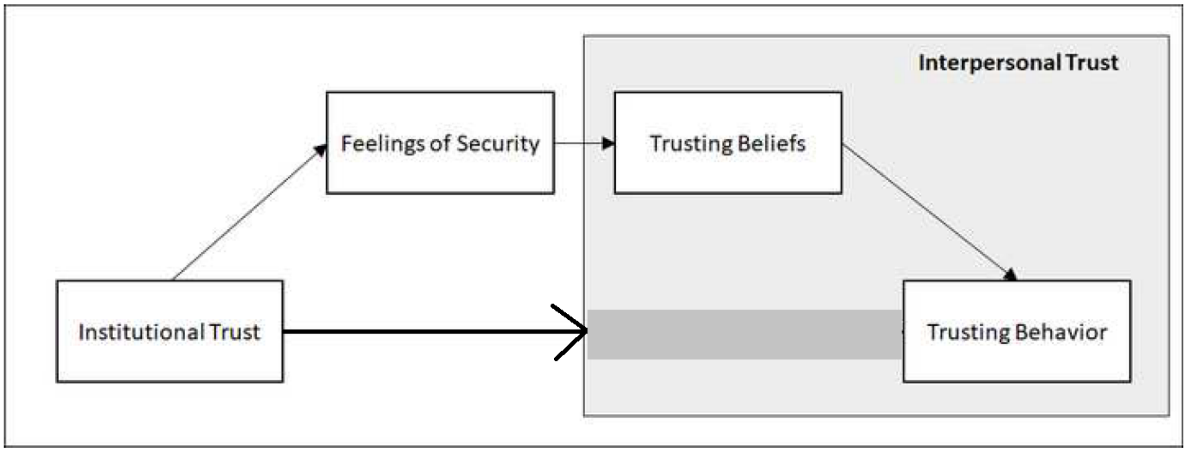

Study 1
The contribution of Study 1 is difficult to grasp at the moment. Indeed, since it does not employ a representative sample of the population and there is no manipulation (not sure if it makes sense to report the results of the sensitivity analysis at p.16), Study 1 appears to contribute very little to the discussion, especially in comparison to Studies 2 and 3. I suggest to move Study 1 to the SI to give more space to Studies 2 and 3, which require more information. If the authors disagree with this comment, I believe they should better justify the value/input of Study 1 (i.e. how does this study exactly improve on our current

knowledge of this relationship?). The following passage at p. 20 provides a good hint on where to start (from my point of view): "This different operationalization of interpersonal trust would allow to relate our findings to previous evidence from survey studies using this scale (e.g., [36]) and to generalize them above specific trust targets (i.e., members of own community such as Italian citizens in Study 1)".

Study 2

1. Given that the mediation mechanism does not involve level 2 (i.e. country-level) variables and they are not interested in exploring the impact of any country-level variables on trust, I don't see why the authors use multilevel mediation. Instead, they could simply control for all country differences (i.e. having countries as a fixed-effect, as they do for survey-years). This should tell us whether the mediation effect is working across countries, which would be more relevant/interesting for their analysis. If the mediation effect is not working once they control for countries, then it might be interesting to understand why this is the case/for which countries the mediation works/what country-level factors are important in this respect (re-introducing the multi-level mediation here).

2. Considering that in Study 3 the authors are manipulating trust towards the police (rather than the broader concept of institutional trust), it would be good to have a separate part of the analysis focusing on the same aspect in Study 2 (trust in the police → feelings of security → generalized trust). This would also help the reader to see more clearly the link between the two studies.

3. As mentioned in the general comments section, I believe that Study 2 needs a table where you concisely present the results from the mediation model (i.e. the different paths for direct and indirect effects) with and without covariates (e.g. Model 1 no controls, Model 2 controls at the individual level, Model 3 controls for countries and survey-years).

Study 3

1. Study 3 requires a more detailed presentation of the design (e.g. how many sessions did you have? How many people per session? It would be good to have more details on the trust game – one-shot? Strategy method? How much could the trusters send? What was the multiplier? Etc.). At the moment, it is difficult to understand what the subjects exactly experienced and in which order (e.g. did they play the trust game after the questions on trusting beliefs? Did the questions on expectations of reciprocity followed the trust game?). This is important to properly evaluate the results of the study.

2. The type of behavioural trust you are measuring here is quite different from the one measured in Studies 1 and 2. Indeed, here subjects are asked whether they would trust someone from another country (i.e. Country X – trustee's home country. Side note: it would be good to know why you had those 11 countries, on which basis you selected them etc.). This is not equivalent to measuring trust towards unknown fellow citizens/strangers, as the form of trust measured in Study 3 involves a stronger out-group component (it should be closer to trust towards migrants). The theoretical framework of Study 3 should discuss this issue and interpret findings accordingly.

3. In my view, deception could have been avoided (by designing the experiment more carefully). I would invite the authors to justify their decision, explaining why deception was needed in this case.

4. Recoding of trusting behavior in Table S4 does not seem consistent with results presented at p.26: “On average, participants transferred 70.6% (SD = 26.7%) of their initial endowment to the trustee, […] (see S4 Table)”. While in table S4 you report the following:

	Institutional Trust	
	Low	High
	M (SD)	M (SD)
Trusting behavior	3.39 (1.39)	3.67(1.28)

How should we interpret the values 3.39 or 3.67 in relation to the value of 70.6%? As mentioned in the general comments, the coding of variables is quite confusing, and it appears to be inconsistent in some passages. Please double-check carefully the manuscript in this respect.

5. Table 2 was cut and didn’t properly show the results.

6. I would like to invite the authors to elaborate more on the mediation effect reported in Study 3. Indeed, while there is no significant direct effect from the manipulation of trust (towards the police) to trusting behaviors, the analysis suggests that there is a significant indirect effect (through feelings of insecurity). Is this a case of indirect-only mediation (e.g. Zhao, X., Lynch Jr, J. G., & Chen, Q. 2010. Reconsidering Baron and Kenny: Myths and truths about mediation analysis.)? Or is it actually due to a moderating effect? Also, how is this consistent with results reported in Study 2 where, in my understanding, we have both significant direct and indirect effects? How do the authors explain this difference? How should we interpret findings from Studies 2 and 3 once taken together?
