# Supplementary material for: Enhancing feelings of security: How institutional trust promotes interpersonal trust
Source: PLoS One. 2020 Sep 11;15(9):e0237934. doi: 10.1371/journal.pone.0237934 (PMC7486136; doi:10.1371/journal.pone.0237934)
Supplement: Attachment — Submitted filename: Response to Reviewers.docx [file pone.0237934.s006.docx]

Dear Dr. Valerio Capraro,

Thank you for conditionally accepting our revised manuscript (PONE-D-20-07477), entitled “*Enhancing feelings of security: How institutional trust promotes interpersonal trust*”, to be considered for publication in *Plos One*. We are grateful for the helpful feedback that the reviewers have provided, and for the opportunity to improve our work.

We carefully addressed the remaining issues in this further round of revisions; we hope that this revised version can solve all the remaining concerns. In particular, in the response letter and in the manuscript, we further clarified some concern raised by Reviewer 1, and addressed the remaining issues pointed out by Reviewer 2.

For an easier navigation of the revisions, our responses are listed in *italics*, and when changes have been made to the manuscript, we insert a section of text from the paper and/or provide the page numbers of the tracked version of the revised manuscript where the revisions can be found. As recommended in the submission guideline, large tables in the manuscript can be fully accessed through the “Web Layout” mode in Microsoft Word.

**Editor**

I have now collected three reviews from the same reviewers who reviewed the first version of this manuscript. One review is positive and accepts the manuscript, another review is also positive and suggests a very minor revision, the third review is still negative, but leaves to the editor the ultimate decision. After reading the manuscript and the reviews, I have decided to follow the majority of the reviewers and conditionally accept this manuscript. Please address the remaining comments at your earliest convenience. I am looking forward for the final version.

*Thanks for conditionally accepting our manuscript. We greatly appreciate the opportunity to further improve the manuscript. We hope that you consider our revisions as raising the manuscript to the level expected at Plos One.*

**Reviewer #1**

Thank you for the opportunity to review this revision. The authors certainly took seriously my concerns and I appreciate their detailed responses. Unfortunately, however, I remain largely unpersuaded by the contribution of this work. It provides arguably interesting data with less than clear findings to a well-researched area in a way that just seems to muddy the water. I must leave to the editor whether this contribution is sufficient.

On the positive side, I buy the authors' argument that the idea that security might explain the institutional/interpersonal relationship is novel but it is not argued particularly well here and as a result, it is hard to see how the paper could stand on its conceptual contribution (the paper does more to say "it could be argued" than to show a concrete logic upon which future, more precise tests can rest).

Additionally, Study 2 appears pretty solid. I am curious about the operationalization of security but I understand that this is secondary data and the argument that the four institutions have some nexus with crime control is persuasive.

*Thank you for your comments on the revised version of the manuscript. We are glad our revision improved your confidence on our findings regarding the contribution of this work and, in particular, the test of the mediating role of feelings of security in the relation between institutional trust and interpersonal trust (especially in light of the evidence provided in Study 2).*

On the negative side, Study 1 still has huge common method variance issues (all three of the focal measures address really similar concepts--the trustworthiness of institutions, whether those institutions create feelings of security, and trust in "most Italians" [which presumably include the people who work for the institutions]). I recognize the attempt to control out these issues and the argument that this is only one piece of a larger puzzle but there still is not much "there" there.

*We understand your concern. However, based on the data and on past research, we are confident that each measure underlies a different construct, and therefore our results are not due to common method variance, for three main reasons:*

(a) *The correlations among those variables are not as strong to suggest that there is a concerning overlapping between constructs (ranging from r = .28 to r = .50; S2 Table, p. 12). Given the correlation between institutional trust and feelings of security (r = .50), we additionally run two confirmatory factor analyses to compare the model fit of either one or two latent factors, to rule out a possible overlap between the two variables and found evidence for a better fit of the two-factors model (see S2 Text);*
(b) *In Study 2 and 3 we used different operationalizations of the constructs and found a consistent pattern of results. For example, Study 2 operationalized feelings of security through a measure that is not tied to institutions, and interpersonal trust with a question that refers to “most people”; Study 3 manipulated (rather than measured) institutional trust and included a measure of trusting behavior (see S1 Table for an overview);*
(c) *These measures are grounded in past research and were operationalized from specific theories (e.g., Yamagishi & Yamagishi, 1994).*

*In the current version of the manuscript, to better clarify the different operationalizations and past research from which we selected these measures, we direct the reader to S1 Table (p. 8).*

Study 3 still has the same issues I brought up in my first review and I was largely unpersuaded by the authors' response. There is still no good reason why a participant would use information about the police in a country where an interaction partner was located to set trust in that individual UNLESS that was literally the only information to go on (as is the assumption here given that this is a manipulation). Thus, this does not represent the actual process within a country and so whatever relation is identified (even if consistent with the one in reality) happened for a different reason.

*We agree with you that there is* “still no good reason why a participant would use information about the police in a country where an interaction partner was located to set trust in that individual UNLESS that was literally the only information to go on”*.*

*That said, this is exactly what we did with our experimental manipulation when we decided not to provide the name of the partner’s country, but now realized this was not explicitly stated in the text. In the current version of the manuscript, we now add two sentences where we explicitly mention this aspect (see p. 21, 22). Hence, participants should base their trust assessments and made their trusting decision based on the information about institutions, as also done in previous research on trust and institutions (see Rothstein & Eek, 2009).*

*Nevertheless, we agree that the ecological validity of the manipulation might not be optimal, but the choice of the experimental method should be understood as a complement to the other methods and data we gathered from different sources.*

Problematically though, that this manipulation still does not result in a direct effect on interpersonal trust remains a problem. I recognize and appreciate the authors' citations of work arguing that this is not a prerequisite of a significant indirect effect so I will stipulate that the indirect effect may exist but the lack of a direct effect supports my contention that the process here is different. We know that institutional trust should covary with interpersonal. That they do not suggests that something is off here.

*We are glad you agree with us that the presence of the indirect effect supports one major goal of the paper.*

*We agree that the lack of a null effect leaves open questions on what could have driven such null effect. However, contrary to what you suggest, the null effect itself is not enough to support specific alternative explanations (as we cannot claim that the null hypothesis is true). As we discuss in the General Discussion section, we believe that there are two potential possibilities which explain these findings. First, in our manipulation of institutional trust in Study 3, the institution does not have a direct intervening role in the game (e.g., the police do not have the power to regulate the social exchange). Another possibility is that the information about the institution learned in this context in light of a one-shot interaction is not comparable to trust assessments toward existing institutions, that are built through recurrent exposure. Both aspects are mentioned in the Limitation and future research section and the discussion of findings of Study 3 (p. 32 and p. 27, respectively).*

*Additionally, we now explicitly mention the lack of a main effect in Study 3 in the overall summary of the findings provided in the General discussion of the revised version of the manuscript (p. 28).*

For smaller issues, the paper still uses Mayer's definition of trust to motivate work on what those authors would call trustworthiness. This just feels unnecessary. As the authors note in the response to reviewers, they root their work in trust defined differently--why not present that as the core definition?

*In the manuscript*, *we refer to the definition provided by Mayer and colleagues (1995) to define interpersonal trust (p. 2), as it is defined as “*[...] the willingness of a party to be vulnerable to the actions of another party based on the expectation that the other will perform a particular action important to the trustor, irrespective of the ability to monitor or control that other party*” (p. 712), and captures well the component related to risk and vulnerability enclosed in trust toward strangers, which is the outcome variable of all the studies presented in the manuscript. Additionally, this work motivated our choice of controlling for trust propensity as individual disposition in Study 1 and 3, as this variable is explicitly acknowledged in the model proposed by the authors. For these reasons, we would prefer to keep this citation unless the Editor has a strong preference about it.*

*That said, we acknowledge that we have used a different definition for institutional trust, based on the work of Devos and colleagues (2002), which is currently presented at p. 5, while discussing research on institutional trust.*

Additionally, the paper would benefit from a read-through for missing words, typos, and word choice.

*Thanks for the suggestion. We made several edits across the entire body of manuscript to improve its readability and address any typo or missing word(s).*

**Reviewer #2**

The authors have addressed very well all points raised, and I believe that the manuscript should be published.
I have only few minor remarks:

- Table 4 (p.26) can still be improved and better show the results for the serial mediation model e.g. results for mediator 2 are not clear (in my view).

*We are glad you found our revision of the manuscript adequate to address most of your concerns.*

*In this revised version, we now clarify how the estimates reported in the table for mediator 1 and 2 were obtained by including a specific remark in the note:*

“*Estimates of regressions of the mediators (feelings of security and trusting beliefs, respectively) predicting trusting behavior.” *(p. 26)*

*We maintained the same structure of the table to be consistent with all the other mediation tables reported in the manuscript, but added an explicit reference to the “serial mediation models” in the heading to differentiate it from the tables summarizing models with a single mediator.*

*We made the same set of edits in Table B in the SI (p. 10), which summarizes a serial mediation as well.*

- The "General Discussion" is sometimes at odds with the "Limitations and Future Research" section. I would invite the authors to double-check for inconsistencies (even if small).The "Concluding Remarks" could be toned down a bit and be reviewed to be more in line with the rest of the manuscript (e.g. this sentence does not fit well with your review of the literature "Decades of research have focused on several processes that may promote trust among strangers, but very little attention has been devoted on one recurrent feature that characterize modern human interactions: the presence of institutions)".

*Following your comment, we revised the three sections of the General discussion section and double-checked the text to remove any inconsistencies among them and the literature review presented in the introduction.*

*For example, we replaced* “(...) the lack of a main effect of institutional trust in Study 3, which does not replicate what we found in Study 1 and 2” *with* “(...) the lack of a main effect of institutional trust in Study 3, which was observed in Study 1 and 2” *(Limitations and Future Research, p. 31), as it might have seemed at odds with claims such as “*That being said, the present findings appear robust and generalizable across research methodologies and variables operationalizations, and remain consistent even when controlling for relevant individual characteristics and institutional performance indicators*” (Limitations and Future Research, p. 32).*

*We decided to completely remove the sentence you mentioned from the “Concluding Remarks” as it looked, indeed, at odds with the content in the abstract and in the literature review. Instead, we wrote a summary sentence coherent with the goals and theoretical rationale as opening of the General Discussion section (p. 27).*

**References**

Devos, T., Spini, D., & Schwartz, S. H. (2002). Conflicts among human values and trust in institutions. *British Journal of Social Psychology*, *41*(4), 481–494. https://doi.org/10.1348/014466602321149849

Mayer, R. C., Davis, J. H., & Schoorman, F. D. (1995). An integrative model of organizational trust. *Academy of Management Review*, *20*(3), 709–734. https://doi.org/10.5465/amr.1995.9508080335

Rothstein, B., & Eek, D. (2009). Political corruption and social trust. *Rationality and Society*, *21*(1), 81–112. https://doi.org/10.1177/1043463108099349

Yamagishi, T., & Yamagishi, M. (1994). Trust and commitment in the United States and Japan. *Motivation and Emotion*, *18*(2), 129–166. https://doi.org/10.1007/BF02249397
